# CP Antenna with 2 × 4 Hybrid Coupler for Wireless Sensing and Hybrid RF Solar Energy Harvesting

**DOI:** 10.3390/s21227721

**Published:** 2021-11-20

**Authors:** Irfan Mujahidin, Akio Kitagawa

**Affiliations:** Microelectronic Laboratory, Department of Electrical Engineering and Computer Science, Kanazawa University, Kanazawa 920-1192, Ishikawa, Japan; kitagawa@merl.jp

**Keywords:** CP antenna, hybrid coupler, wireless sensor, hybrid RF solar, energy harvesting

## Abstract

The main challenge faced by RF energy harvesting systems is to supply relatively small electrical power to wireless sensor devices using microwaves. The solution is to implement a new device in a circularly polarized rectenna with circular polarization sensitivity integrated with a thin-film solar cell. Its dual-feed antennas are connected to a 2 × 4 asymmetric hybrid coupler and a multi-stage voltage doubler rectifier circuit. This configuration has a 2 × 4 asymmetric hybrid coupler used to produce 4 outputs with a 90-degree waveform phase difference. The two ports can independently be connected to the wireless sensor circuit: radiofrequency harvesting of hybrid energy solar and information equipment can be carried out with these two antennas. The Dual-Feed circular patch antenna has a two-port bandwidth of 137 MHz below −15 dB and an axial ratio of less than 3 dB, with a center frequency of 2.4 GHz with directional radiation and a high gain of 8.23 dB. It can be sensitive to arbitrary polarization of the input voltage multiplier waveform to overcome uncertainty in empirical communication environments. A parallel structure is arranged with a thin film solar cell integration from the transmitter with an output voltage of 1.3297 V with a compact composition and RF energy. The importance of adopting a wireless sensor strategy with circular polarization sensitivity and integrated RF solar energy harvesting rather than a single source method makes this research a significant novelty by optimizing the analysis of multiple wireless sensor signal access.

## 1. Introduction

With the high demand for communication devices, the challenge of providing wireless sensor devices without batteries increases, along with the need to significantly reduce replacement costs and battery waste. A Rectenna is a transducer device whose primary function is to convert RF (Radio Frequency) energy into electrical energy. It performs as a wireless battery to supply wireless power continuously for these portable devices in positions far from each other [1,2], or by utilizing wasted scattered RF or electromagnetic waves in the air. The integration of rectennas, solar cells, and Wireless sensor devices in communication systems allows the development of new technologies for wireless energy harvesting and information transmission simultaneously without interference. Therefore, the configuration concept in this article is Wireless Sensing and Simultaneous Power charging independently, which has provided a solution to the problem and presents significant new value in the future for wireless communication systems.

Several studies on antennas that utilize a hybrid coupler have been published to develop a wearable communication system for wireless sensors and data communication transmission [3,4]. In addition, improvements in developing optical energy materials are increasingly efficient in harvesting solar energy [5]. The antenna configuration utilized has one port and two ports for the feed couple, based on previous research. The antenna is utilized for a single port system for wireless communication and transmitting power to the rectenna. However, the rectennas of one port influence each other between the two functions because they are utilized interchangeably [6,7] so that they affect information changes. Recently, several two-port rectennas for data communication transmission and wireless energy harvesting have been designed [8,9]. The addition of the optical element integration in solar cells has been studied to optimize the optical and electrical properties of the most promising examples [10]. In [11], a microstrip rectenna between two ports is designed for data communication transmission and energy harvesting. However, the rectenna applied configuration in previous research has a complex multi-layer structure, and the resonant frequency is different for each feed port, limiting the application scenario. Antennas with microstrip circuits with dual feed channels and two alternative ports are proposed to provide solutions, that is, for energy harvesting to achieve typical harmonic frequency bands for radiofrequency harvesting and wireless sensors. It can accommodate both energy sources: solar cells and RF independently and independently of the performance of the wireless sensor system and energy harvesting and without changing the transmission process. Of course, this process uses a compact configuration and inexpensive materials. Another advantage is that the analysis applied to the antenna utilized a powerful method of multi-signal classification for multi-input and multi-output communication networks.

## 2. Design Circuit Structure

**Figure 1:**
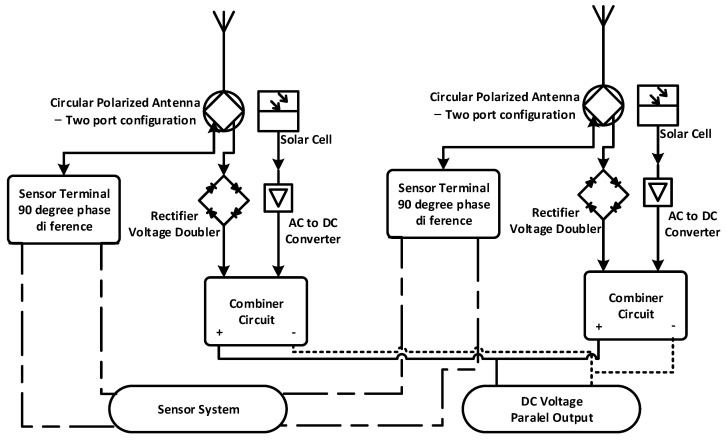
Schematic of the CP Antenna with a 2 × 4 Hybrid Coupler.

Figure 1 shows that the proposed configuration can adopt two rectenna structures integrated with thin-film solar cells. It has circular polarization sensitivity and an asymmetric 2 × 4 hybrid coupler to produce 4 different wave outputs on the coupler element as an RF combiner circuit with every two sides 90 degrees at synthesizing wave transmission on a wireless sensor antenna.

The configuration scheme consists of two main components as the design structure. It is a Dual Port CP Antenna that functions as a collector of RF waves energy that has been integrated with solar energy harvesting media. Other main components are a 2 × 4 Direction-Finding Hybrid Coupler and Hybrid Electromagnetic Solar Circuit as a rectifier of energy from an AC source, and a passive component of a wireless sensor directly connected to the Dual Port CP Antenna.

### 2.1. Dual Port CP Antenna

**Figure 2:**
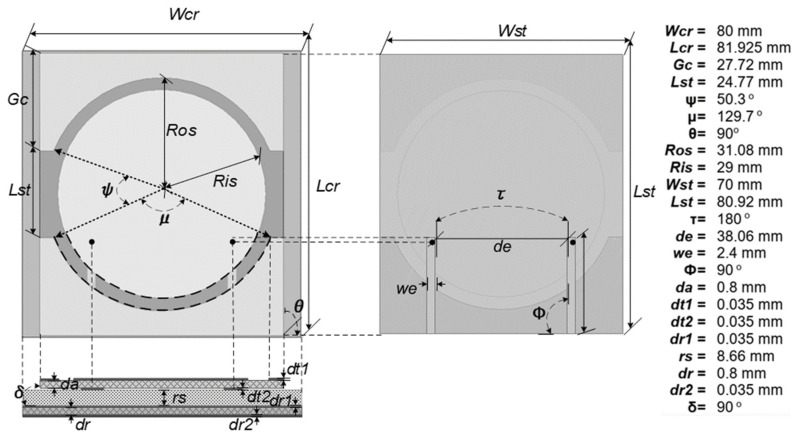
Double slot with dual feed CP Antenna Geometry.

The CP antenna design is based on a circularly polarized patch antenna. Optimization of the circular patch is the feeding probe from the center of the patch to the disk side area of the LHCP (Left-hand Circular polarization) and RHCP (Right-hand circular polarization). The orientation of circular transmission wave portions and double slot U increases the antenna’s sensitivity to circular polarization waves. This change ensures that the antenna in Figure 2 has symmetrical and similar performance in each mode of the feed working frequency. Figure 2 also shows the ideal circular double slot U circular patch antenna case in a circular polarization operation. It is sensitive to wireless sensors’ horizontal polarization (HP) and vertical polarization (VP) antennas. The dual-slot U slot is constructed on a circular surface with the open slots in the direction of the side concentrically via the feed antenna. An antenna patch setup is connected with a circular polarization aperture. The antenna consists of two layers, with a phenolic white paper antenna substrate with the intrinsic specifications of the material used, which is generally used as a reference for determining the dimensions of the transmission line in the feed line circuit (*ε_r_* = 4.2, tanδ = 0.0018, h = 0.0035 mm) with a two-port patch antenna installed and a 50 Ohm SMA port utilized for network feeding. The FEM (Finite Element Method) analysis used to solve numerical differential equations for 3D electromagnetic modeling in the High-Frequency Structure Simulator Software is utilized to optimize the implementation and design of the structure.

The direction of wave polarization is related to the via hole orientation on the feed as a medium for propagating waves from the main patch. With this configuration, the linearly polarized antenna feed position can be reconfigured via disk surface to control one polarization orientation in the electromagnetic design TM_11_ mode [12,13]. The antenna configuration has a symmetrical geometry; therefore, the antenna’s electrical characteristics in the two feed states have the same behavior in orthogonal polarization. Briefly, the working frequency of the antenna can be represented by Formula (1) to determine the position of the feed circular polarization.

(1) $$\frac{E_{y}}{E_{x}}\approx \;\frac{\sin\left(\frac{\pi }{2R}y^{\prime }\right)k^{2}\left(1-j/Qt\right)-{\left(k_{x}\right)}^{2}}{\sin\left(\frac{\pi }{2R}x^{\prime }\right)k^{2}\left(1-j/Qt\right)-{\left(k_{y}\right)}^{2}}$$

**Figure 3:**
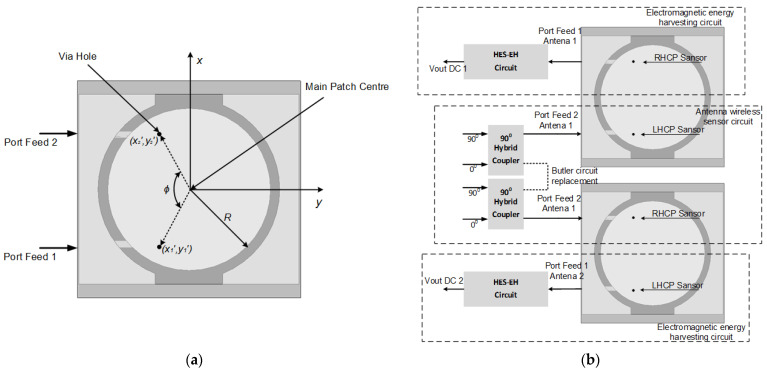
(**a**) Arrangement via the hole on transmission feed (**b**) Feed transmission on CP antenna configuration.

Thus, feeding the element along the disk side position starts from the angle, according to Figure 3a. It is indicated by the dashed line resulting in an ideal left circular polarization on the broad side of the circular polarization that can be obtained if the E field ratio is 3 dB [14]. To represent the position of the via hole to the axial ratio value, therefore decomposed into *E_y_*. It is an E-field linear polarized to the *y* axis. Additionally, *E_x_* linear polarization to the *x*-axis to produce a right-hand circular polarization can be achieved by feeding along the opposite radius, which starts from the lower right corner and continues to the upper left corner to produce a left-hand circular polarization. This method obtains circular polarization to place the via hole in a feed based on optimization. Thus, based on the optimization results, the via-hole position formulation for the antenna design is obtained by Formula (1).

The value in Formula (1) of *Qt* = 1/tan δ, and the *k* = (*k_y_* − *k_x_*) − *Qt*. Tan δ is the loss tangent of the material. Based on the optimization, the angle ϕ separates the two via holes is determined. It is obtained to produce an orthogonal field to each other under the patch and outside the patch. The via hole is positioned at the point where the other via-hole generates the field; therefore, the reducing effect significantly changes the performance between ports to increase the antenna’s sensitivity to wave polarization [15]. The ground plane separating the primary patch connection and the double U slot is smaller than the resonance size so that radiation has propagated to the main patch element. Assuming the circular patch resonates in its dominant mode, the accumulation of the slot optimization model concerning the frequency formed on the patch surface is expressed in Equation (2).

(2) $$f\approx c{\left(\varepsilon_{r}+1\right)}^{\frac{1}{2}}\;{\left\{\pi \;R_{is}\;{\left(2\varepsilon_{r}\right)}^{\frac{1}{2}}\right\}}^{-1}$$

**Figure 4:**
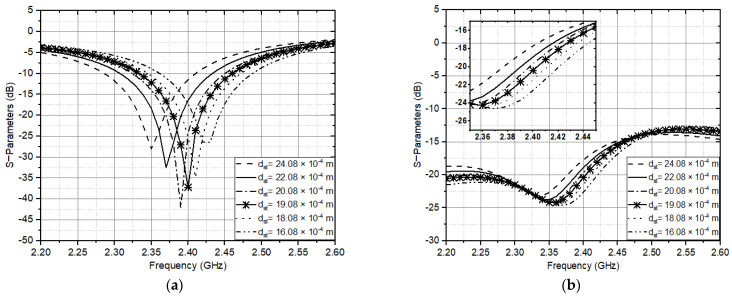
Arrangement S parameter calculation to the dimensions of the double U slot on (**a**). S11 on port 1 (**b**). S21 on port 2.

The speed of light as *c* in free space with a value of 3 × 10^8^ m/s and *ε_r_* is the permittivity value of the antenna substrate, which is the phenolic white paper. The resonant frequency at each output port on the CP antenna is 2.4 GHz, which has the same resonant frequency with a lower axial ratio. Using the formula to estimate the operating frequency of the two ports to produce CP axial ratio, the double U slot is optimized, and the slot width calculation dst = *R_so_* − *R_si_* to determine the path distance to the ground. With a tolerance value of less than 4% when the slot width is 0.0001 m. The double U slot in the design has the advantage that most of them independently have a resonant frequency on the antenna with a specific band range on the performance of other antennas with changes in the frequency value in Figure 4a,b. Figure 4b has a lower slope point than Figure 4a. It is due to the wave reflection on port 1 in Figure 4a. However, it does not significantly affect the antenna’s resonant frequency.

However, the position and dimensions of the double U slot must always be symmetrical between the top side and bottom side on the entire patch surface so that every shift in the resulting polarization value still has circular sensitivity. Of course, the essential circular polarization adopts an electromagnetic configuration via hole according to the optimization in Equation (1), the incident wave that propagates to the feed antenna and the reflection from the reflector.

Tuning the resonance frequency with double U slot optimization (d_st_) based on the radius of the circular patch dimension to the ground distance obtained from d_st_ = *R_so_* − *R_si_* with a value of d_st_ with a shifting accuracy up to 10–5 mm at the narrowband frequency so the correct frequency capture on the antenna settings according to the implementation.

### 2.2. 2 × 4 Direction-Finding Hybrid Coupler and Hybrid Electromagnetic Solar Circuit

**Figure 5:**
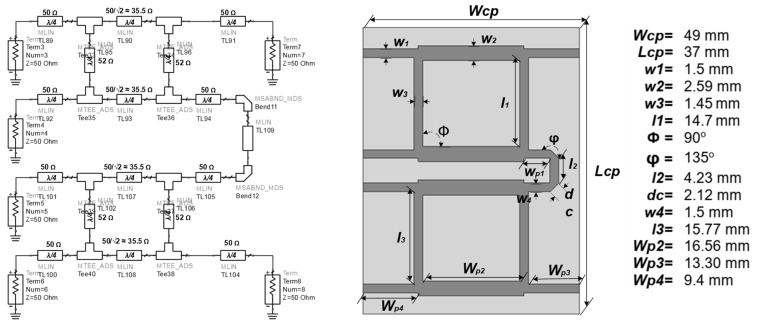
Stub Geometry on the 2 × 4 Direction-Finding Hybrid Coupler.

The circuit consists of two feed functions on the CP antenna. The first function is to transmit sensor signals, and the second is to convert electromagnetic and solar energy into electric energy. The sensor signal transmission circuit consists of a hybrid coupler that produces a 2 × 90 degree phase difference. At the antenna line transmission to the hybrid coupler feed, the two antennas are connected to a couple that has a 90-degree phase-shift that is coupled to each between two output feed lines in an orthogonal position. The first sub-strip is a straight configuration feedline with a dimension of λg/4, and the wavelength is expressed as g in the waveguide. The next branch contains a microstrip with four-line feeds with dimensions of about 3λg/4, and the branching pairs are manipulated to have compact dimensions [16,17]. Then, the wave is transmitted via tuning stub optimization to the second coupler branch as a reference guide for the second hybrid coupler, physically listed in Figure 5.

**Figure 6:**
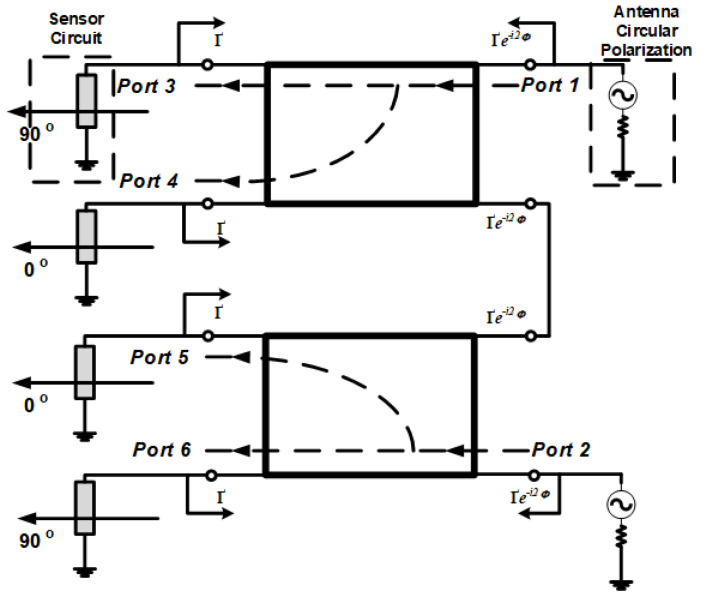
Wave propagation on the 2 × 4 Direction-Finding Hybrid Coupler network.

The dual hybrid coupler construction uses a stripline identic with two rectangular circles with eight stubs configured as six ports. This construction consists of two sub-phase shifters, and each sub consists of four feed stubs with one bend on the right side between ports 1 and 2 in Figure 6, which are indirectly connected to the two CP antennas. There are four feed coupler ports on the left side with port 6, port 5, port 4, and port 3 as a connecting strip to the wireless sensor network with the media feed towards one of the connecting stubs between the hybrid coupler.

The proposed microstrip antenna has circular polarization. The coupler circuit is connected to the antenna when the antenna only operates on one 50 ohm port based on effective impedance. Additionally, the port as receiver energy is fed to a 50-ohm load with a suitable alternative load Z_L1_, which has a complex impedance assuming that the loss coupler is negligible [7,18]. Suppose the circuit impedance between the feeds does not match from the coupler side. In that case, the reflected wave from port 4 is 1 so that the reflected wave at port 3 is *jҐα*1 where *Ґ* is the reflected wave coefficient on the wave propagation in the stripline hybrid coupler and α is the representation of the signal originating from port 4 which propagates with the reflected wave. *β* is the propagated reverse wave on the circuit with the numbers following, which is the port shown in Figure 6. Then, the signal is transmitted back to ports 4 and 3 through the coupler; therefore, the output waveform on the reflection coefficient at port 1 is from port 3 in the form of *β*_3_ = −*jҐα*_1/_*√*2 (*β*_3_ = −*Ґα*_1/_*√*2) and on port 4 *β*_4_ = −*Ґα*_1/_*√*2 (*β*_4_ = −*jҐα*_1/_*√*2) or the incoming feed-side coupler where 1 is the input waveform of port 1. Then, the reflection value can be expressed as *α*_3_ = *jҐҐe^*−*i*2*^^^Φ^^α*_1/_*√*2 (*α*_3_ = *ҐҐe^*−*i^*^2^*^^Φ^^α*_1/_*√*2), and *α*_4_ = *ҐҐe^*−*i^*^2^*^^Φ^^α*_1/_*√*2 (*α*_4_ = *jҐҐe^*−*i^*^2^*α*_1/_*√*2), which propagates to the coupler and then to Port 1 with *Φ* as the phase length of the propagated wave; on the feed, the coupler path propagates to the other side. The output wave propagation is *β*_1_ = 0 (*β*_1_ = −*jҐҐe^*−*i^*^2^*^^Φ^^α*_1_) and *β*_2_ = −*jҐҐe^*−*i^*^2^*^^Φ^^α*_1_ (*β*_2_ = 0). *β*_1_ and *β*_2_ are the reflection coefficient values for ports 3 and 4. At a reflection value of 1, where one of the feeds goes towards the other side, the value of the matching circuit causes the deficiency of a reflected wave based on Port 1; it can be expressed as *β*_1_ = 0 after reflection occurs. This happens when no reflected wave returns to Port 1.

However, at *jҐα*1, there is no reflected wave from the feed through to the other side of the coupler, and arbitrary circuits can reuse the reflected wave *β*_1_ = −*jҐҐe^*−*i^*^2^*^^Φ^^α*_1_. The feed structure differs from the antenna. The feed structure varies in input power and output load. However, this can work well as long as it can remain impedance-matched. So that there is no reflected wave, the wave structure can be reused even to increase the efficiency of the shifting conversion of the reflected wave.

**Figure 7:**
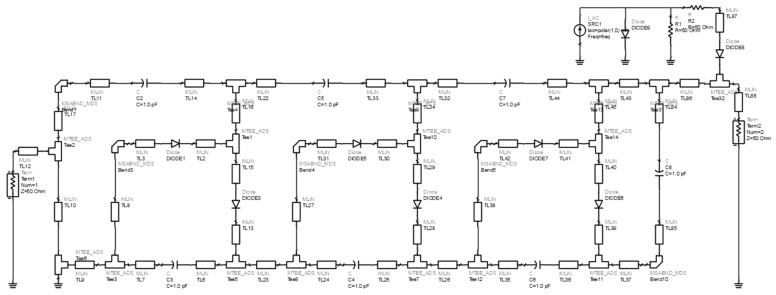
Configuration of the 3-stage voltage doubler circuit.

In a narrow local area, power transmission on radio frequency with high input power density is prohibited by regulations for health reasons. According to these rules, to meet the safety standards for transmitting RF waves, they must have a power density (*σ*) of less than 1 mW/cm^2^ [19]. Based on these regulations, the circuit design in this study uses a power rectifier with an intermediate input. The circuit design configuration is described in Figure 7. The circuit configuration design is printed on the FR4 substrate, which is identical to the antenna with a coplanar structure. The components in the rectifier circuit design include capacitors, series rectifier diodes, and circuits with a 3-stage voltage doubler configuration.

Low-loss rectifier diodes are a significant concern in achieving a high AC to DC voltage conversion value at <1 mW/cm^2^. Based on previous high-frequency research, rectifying is effective at a low threshold voltage (Vbi = 0.25 V); a single SMS 7621-079LF Schottky diode is utilized, with a bias capacitance value (Cj = 0.18 pF), which is connected in series [20,21]. It is based on the working principle of a multi-stage circuit doubler. In addition, the advantages of diodes in circuit design are that they have low power consumption and band switching at a frequency of 2.4 GHz according to antenna specifications. Optimization is additionally carried out. Line feed dimensions and capacitor values are based on the calculation of the electrical circuit. Then, by electromagnetic method analysis, the wave propagation is simulated using ADS software with an impedance following the feed on the antenna and thin-film solar cell with the circuit source scheme shown in Figure 7.

**Figure 8:**
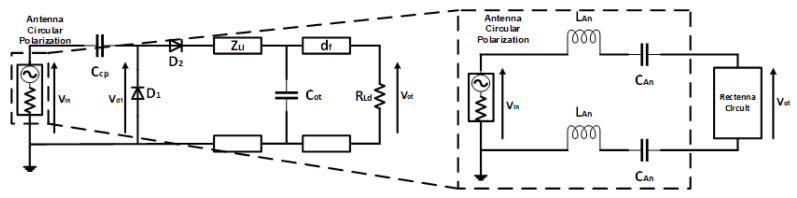
Equivalent Circuit of the CP antenna Energy Source.

The equivalent circuit of a rectenna with a voltage doubler configuration using the Dickson charging pump model is shown in Figure 8. Two Schottky diodes, D_1_ and D_2_, are placed in series and shunt circuits in the impedance circuit configuration. Then, to provide a long-lasting effect on the delivery of the waveform, a capacitor is embedded between the transmission lines with a C_ot_ value, each with an impedance value of Z_li_ and df with a DC output load at a load of R_l_.

The circuit design’s optimization did not have a capacitor charging pump with isolation of the ground on the rectenna on one side of the antenna branch because the DC voltage does not propagate through the antenna. However, this configuration is ineffective if utilized on an antenna connected directly to the ground, such as occurs with antenna Loops [22,23,24]. Thus, the source of electromagnetic energy at an angular frequency is the power input of the rectenna. In the circuit configuration, the source impedance is designed at 50 Ohm impedance, with the same value as the antenna impedance. The antenna dimension design for impedance is calculated with electromagnetic methods analysis using 3-D High-Frequency Structure Software with a normalization description on the Smith chart. The input power *P_if_* can be described using Equation (3):

(3) $$P_{if}=\frac{\lambda^{2}}{4\pi }G_{r}W_{r}$$

where *λ* is the wavelength, *G_r_* is the gain of the receiving antenna, and the power density in the receiving environment is expressed as *W_r_*. The value of *G_r_* is the antenna gain according to the design configured with the circuit. When measuring the rectenna, the *W_r_* value is tuned to 1 mW/cm^2^ so that, at a distance of 30 cm, the *P_if_* value is 10.28 dBm. The analysis of the antenna circuit design can be represented in an equivalent circuit as a resonance circuit at the antenna’s instruction load value. The inductance is *L_An_* and capacitance *C_An_* in Equation (4). Adjustments the resonant frequency, which must meet Equation (2) by *ω* for generating *f*, the value of which is utilized later for process analysis [25,26] on the propagation of the transmitted RF wave.

(4) $$\omega ={\left(L_{An\;}C_{An}\right)}^{-\frac{1}{2}}$$

From the antenna equivalent circuit implemented into line w, therefore, in the equivalent circuit, the antenna only works in the AC voltage domain so that the rectifier characteristics are not affected by *L_An_* and *C_An_*. However, to guarantee the minimum capacitance, the *C_An_* must be smaller than the charging pump capacitor. The *L_An_* and *C_An_* values are obtained as 6.5 nH and 0.65 pF by ensuring that no electrical charge is stored on the Can at a frequency of 2.4 GHz.

## 3. Performance and Analysis

### 3.1. Double Feed CP Antenna—Double U Slot

**Figure 9:**
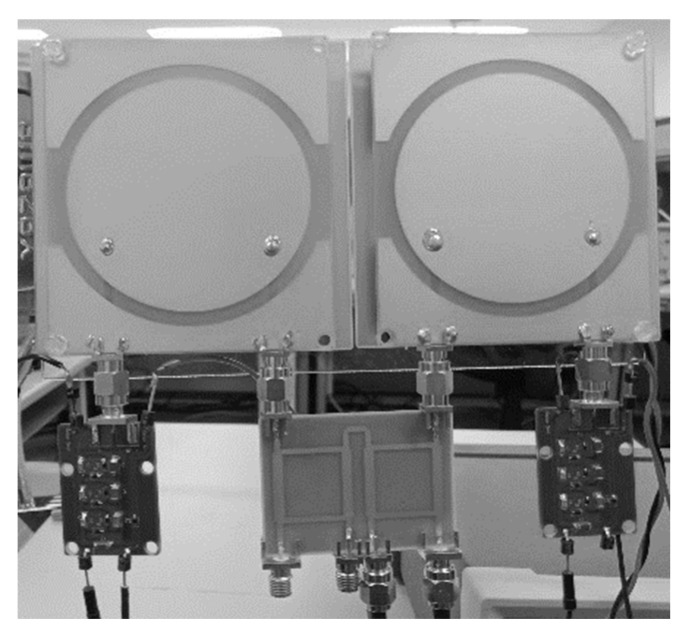
Implementation of the prototype CP Antenna with 2 × 4 Hybrid Coupler for Hybrid Electromagnetic Solar Energy Harvesting and wireless sensor.

The depiction of a microstrip antenna has a circular polarization. Therefore, the analysis in this work considers several critical antenna parameters. The vital parameters include the proposed antenna performance of the via hole radian area position of the antenna patch and the dimensions of the shaped slot around the patch. Figure 9 describes the complete circuit implementation.

**Figure 10:**
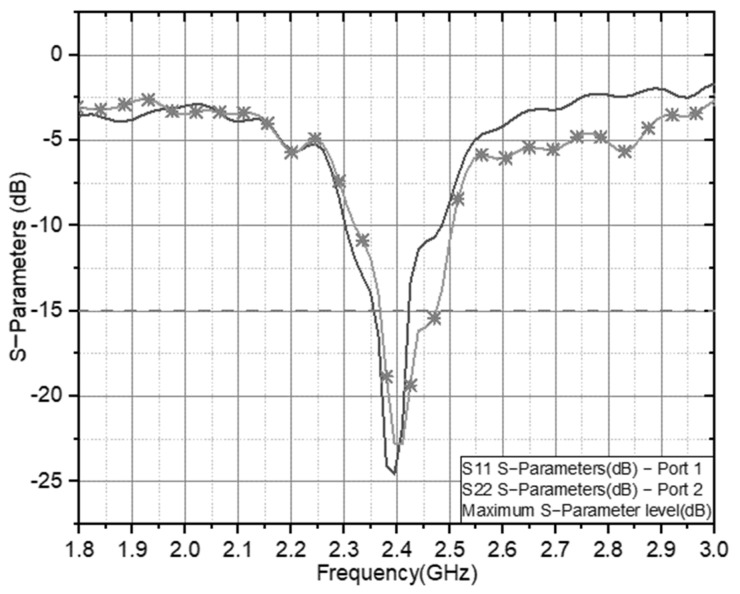
S Parameter of the Double U slot with a dual feed CP Antenna.

One optimized antenna parameter is the dimension of the double U slot, which is directly affected by the circular patch radius. The antenna ground also influences on the axial ratio for CP and impedance matching for CP antennas. The antenna setup was measured using a Rhode and Schwarz ZVL Network Analyzer 9 KHz–13.6 GHz, and Figure 10 presents the results of the S parameters calculated for the antenna. It describes that the antenna with double U slot Dual Feed has a balanced operating frequency band, and the resulting bandwidth is about 137 MHz (12%) at a focus working frequency of 2.4 GHz (2.35–2.47 GHz).

**Figure 11:**
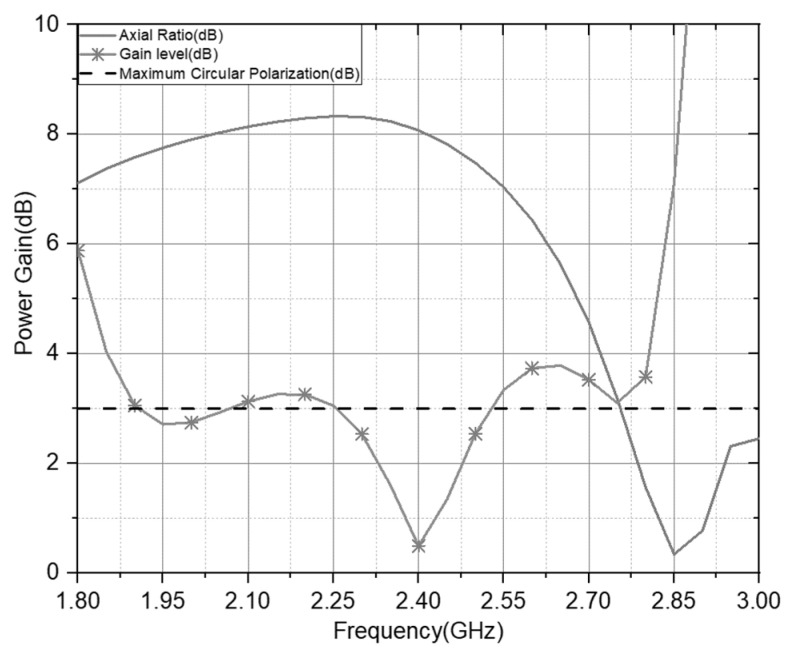
Power Gain level and Axial Ratio of the Double U slot with the dual feed CP Antenna.

The axial ratio and gain on the antenna performance at the 2.4 GHz resonance frequency are shown in Figure 11. The antenna offers comprehensive coverage at the working frequency with AR < 3 with a gain value of 8.23 in the targeted range area. It has an effective bandwidth of 24%. Therefore, the dual-feed structure with a double U slot can accommodate the impedance bandwidth, circular-polarization characteristics, 3-dB beamwidth with a dual feed method, and antenna material with the low-cost profile. This research proposal is based on previous research [27,28], showing that the proposed antenna has a directional, compact slot structure and a 3 dB wide beamwidth. The antenna [29,30] has a compact dimension of the proposed antenna, however it does not have double CP radiation. Therefore, the compact dual-slot CP antenna is more suitable for integrating wireless sensor devices for long-range applications than other antennas.

**Figure 12:**
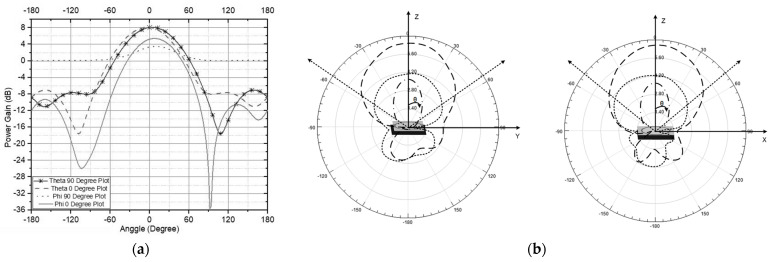
Radiation Pattern of the Double U slot with the dual feed CP Antenna (**a**) Rectangular plot (**b**) Radian Plot.

Figure 12 shows the antenna radiation pattern performance in the elevation plane (φ = 0°) and the azimuth plane (θ = 90° and 0°) for an antenna working at 2.4 GHz, which is shown in Figure 12a with a rectangular plot. To describe the level scattering power and circular property, Figure 12b shows the antenna’s electromagnetic wave distribution area pattern. The antenna radiation has a single beam pattern with a maximum elevation field of ±87°. The azimuth plane has a directional radiation pattern with a side lobe of less than 0.9 dB. However, in the elevation plane (θ = 90°), there is a loss of pattern directionality caused by the grounding effect on the antenna.

### 3.2. Rectifying Multistage Hybrid RF Solar Energy Harvesting and Wireless Sensing

Due to its symmetrical configuration between the antenna ports and the reciprocal of the two couplers through a two-way connection between the antennas, the placement of the rectifier circuit can be switched independently without disturbing the communication channel, as shown in Figure 3b. Moreover, the proposed hybrid electromagnetic solar rectifier circuit is flexible. It was shifted to be optimal according to user’s needs [31,32]. As the hybrid electromagnetic solar rectifier circuit works on the same frequency band or the same frequency band on each low band port, each port shares the power with the same value.

The input wave coming from the antenna has a circular polarization sensitivity and mismatch factor *v* = 0 on one of the antenna ports that deliver the power or signal in the process of transmitting data communication which propagates through the hybrid coupler as a wireless sensor. Thus, in the equivalent circuit calculation, the antenna port and the hybrid coupler are connected to a 50-ohm load with matched and isolated conditions. There is no power loss on the load circuit of 50 ohms. Therefore, the rectenna and communication circuits can receive the overall RF power without any wasted power-sharing with efficient circular polarization matching. Then, at a predetermined frequency (*f*), the input power of the rectenna circuit is therefore expressed as:

*P*_*Rx*_ (θ, φ, *f* ) = *vP_r_* (θ, φ, *f*)η*_a_* (*P_if_*) (5)

The power received by the antenna is expressed by *Pr* (θ, φ, *f*), and the input power of the rectifier circuit is defined by (*P_if_*), which is correlated with the arrival angle (θ, φ, *f*) as an indicator of the transmission process in the communication system. In addition, to express the efficiency of the antenna based on the frequency function, it is represented by η*_a_*.

**Figure 13:**
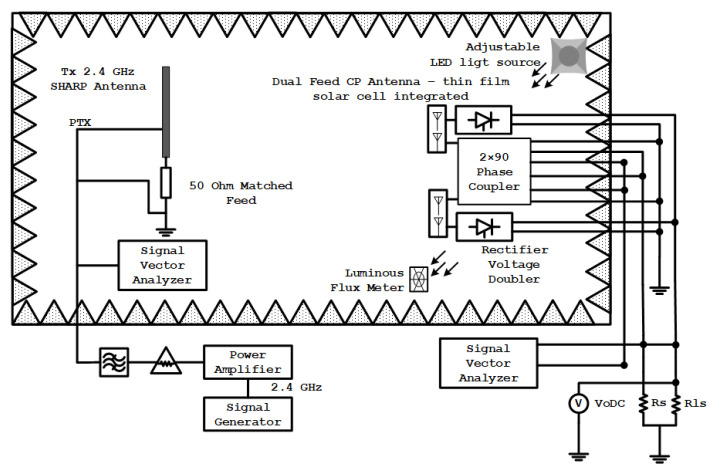
Configuration of the prototype circuit performance test.

With two antenna ports with identical configurations between the two feeds, the communication circuit and rectifier can work independently with equal or different working frequencies. Still, they must be in the antenna operating frequency range and the bandwidth of the couplers between 2.35 and 2.47 GHz. The configuration of the prototype circuit performance test is according to Figure 13. The signal generator generates the signal of the transmission device with an operating frequency of 2.4 GHz. Signal amplification and filtering are carried out with predetermined variables and monitoring of signal structure with the signal vector analyzer. The receiver setup is carried out from the RF signal-received process by measuring the output voltage using a high precision multimeter and a signal vector analyzer for the communication system parameters. Additionally, the measurement of integrated thin-film solar cells is carried out using an adjustable light LED source that can spread light evenly in the room and then measuring the light luminance in the room using a luminous flux meter to determine the amount of luminous light that is spread by the LED and exposed to the thin solar cell file.

Based on the configuration in Figure 13, it has been confirmed that the rectenna circuit independently has not affected the signal injection process leading to the hybrid coupler. Therefore, the efficiency of RF-DC power conversion is optimally utilized without any interference in the communication system, where (η*_r_*) is the RF-DC conversion efficiency which the following equation can express:

(6) $$\eta_{r}=\frac{V_{Out}/Z_{L}}{P_{Rv}}\times 100\%$$

With the power received by the rectifier circuit from the *P_Rv_* antenna, the load impedance is expressed by *Z_L_*, and the DC output voltage above the load is defined by *V_Out_*, thereby comprehensively identifying the DC output voltage in each circuit configuration. Before measuring the *V_Out_* of *P_Rv_* performance, this is done by testing and taking several power samples that the antenna can obtain in far-field conditions with the power spectrum level on the signal vector analyzer, which is represented on the spectrum in Figure 5.

**Figure 14:**
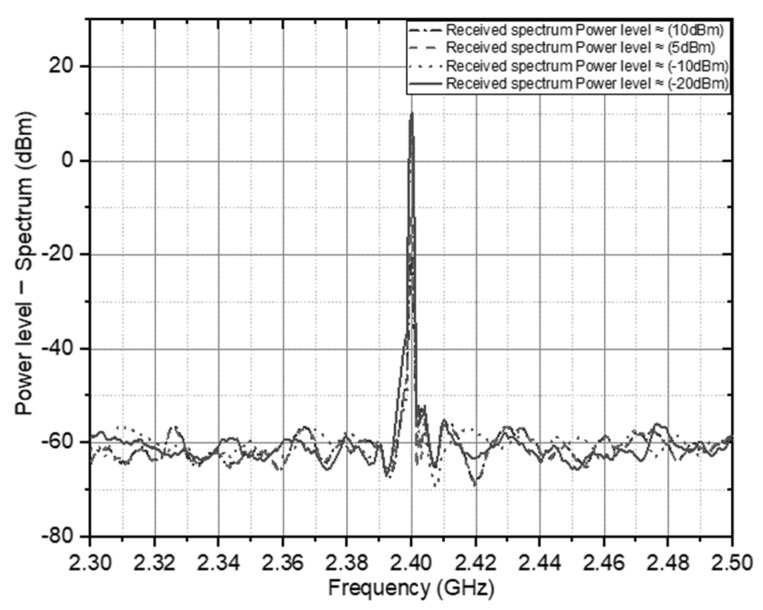
Spectrum graph of the signal reception level by the CP antenna.

**Figure 15:**
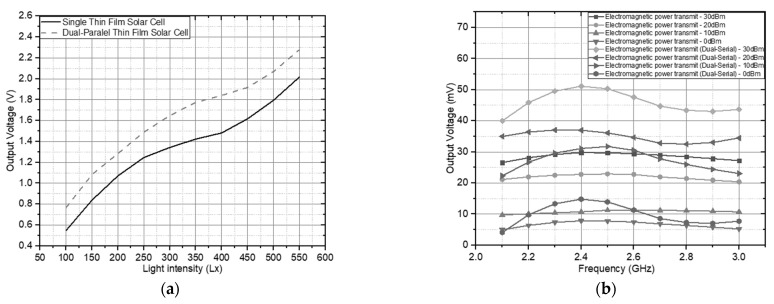
Rectenna output voltage from the source: (**a**). thin-film solar cells; (**b**). dual feed CP Antenna.

The electromagnetic wave transmission spectrum level is calculated into four sample levels to represent the reference antenna working at the 2.4 GHz frequency. With a spectrum of transmission levels of 30, 20, 10 and 0 dBm, with the spectrum received in Figure 14, the spectrum is adopted as a whole, covering the transmission of empirical scattered electromagnetic waves. In the configuration carried out, the first solar energy test, which has been integrated without involving electromagnetic energy, is carried out to identify the ability of thin-film solar cells in the rectifier circuit. The process of generating solar energy in the configuration requires a certain level of light so that the voltage can flow in the circuit performance. Figure 15a shows the voltage level on the light luminance on exposure to the solar cell.

After testing the solar cell, the RF circuit was then tested in transmitting RF energy. It was spread with an isotropic power emission model with a maximum effective radiation of 30 dBm, applied to the experiment in Figure 15b. In the experiment, the main focus was on the variation of the emitted power to obtain a voltage as a representation of power based on Equation (5), following these conditions if it is to be practically implemented.

**Figure 16:**
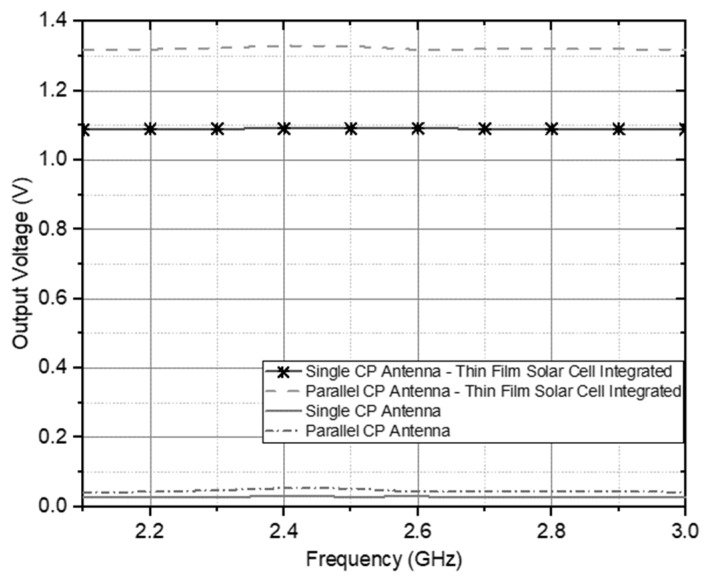
The increased voltage on the integration of the CP Antenna with the 2 × 4 Hybrid Coupler for Hybrid Electromagnetic Solar Energy Harvesting.

Performance measurement produces a DC voltage based on various power transmitted at an operating frequency of 2.4 GHz on an antenna measured using a spectrum analyzer stated in the spectrum level shown in Figure 14 under ideal conditions. Figure 16 confirms that increasing the power level in the spectrum received by the antenna provides an increase in the DC output voltage. According to the circuit specification, the increased output voltage is higher at the resonance frequency value of 2.4 GHz than the other frequency resonance values. The DC output voltage obtained at 2.4 GHz is approximately 54,344 mV recorded at the 10 dBm level spectrum. Thus, the practical circuit can work better as an electromagnetic energy harvester than [33,34,35]. Then, to increase voltage levels, higher performance measurements are integrated with thin-film solar cells.

Furthermore, the maximum conversion efficiency, calculated according to Equation (6), is achieved at the circuit’s intrinsic resistance for a given transmission power. The rectifier with the medium input power of −10 to 10 dBm has a value of RF to DC conversion efficiency of 51.5%, and the high power is 30 dBm with an efficiency level of 53%. It concluded that the conversion efficiency would increase if the transmitted power was increased because the multi-stage doubler this work designed operates at a low current level, emphasizing optimization of the output voltage. The separation between the two feed antenna elements in Figure 16 shows the DC voltage output in integration with a thin-film solar cell with a significant maximum increase of 1.3297 V on 200 Lx. The performance comparison of previous research and development configurations of energy harvesting and wireless sensing can be analyzed in Table 1. In Table 1, after comparing previous studies, the proposed circuit configuration has one main clear advantage: its configuration ability to communicate and harvest energy simultaneously or as an independent operation with two ports with circular polarization sensitivity and a higher energy conversion efficiency value than configurations proposed in previous studies.

**Figure 17:**
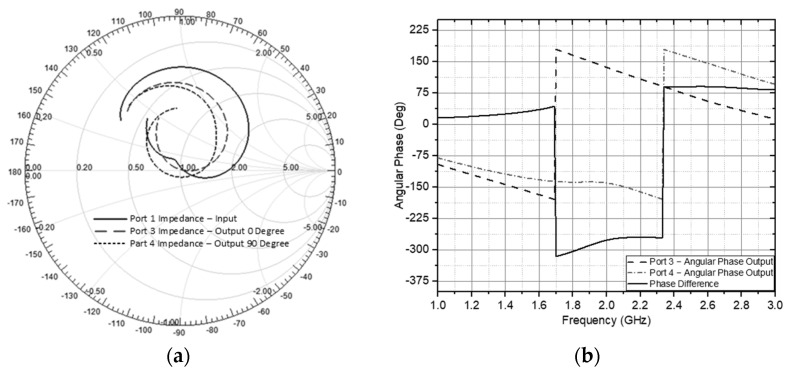
(**a**) Hybrid Coupler circuit impedance (**b**) Hybrid Coupler circuit phase output.

In the wireless sensor system, the hybrid circuit coupler has its main parameter in the implementation function. It produces a phase difference of 90 degrees for each of the two output ports in the four output ports with an impedance according to the circuit qualification, namely 50 Ohm as shown in a comprehensive analysis in Figure 17b. The wave phase value based on the hybrid coupler output is symmetrical so that one side can be taken as a reference. It is between ports 1, 3, and 4 because the other side has identical values. At the output of port 3 = 80.6281° and port 4 = 170.5510° so the value of the phase difference = 89.8227°. The phase difference is still efficient in the communication circuit even though it is not exactly 90 degrees because it is still within the tolerance of ±5 degrees. Then, the impedance of the circuit performance is described in the Smith chart as a measurement model in Figure 17a at port 1 = 1.0101 + 0.0153i, port 3 = 0.9732 + 0.0829i and port 4 = 0.9809 − 0.078i. From this value, the value 1 is normalized as 50 ohms at Z impedance. Based on the impedance performance value, it is close to 50 ohms and, according to tolerance, reflected waves do not therefore have a significant effect on the transmitted and received signal. With these specification parameters, the overall configuration can work well on the adaptive antenna system, which is then described in the configuration analysis in the sensor wireless communication system in the next sub-section.

**Figure 18:**
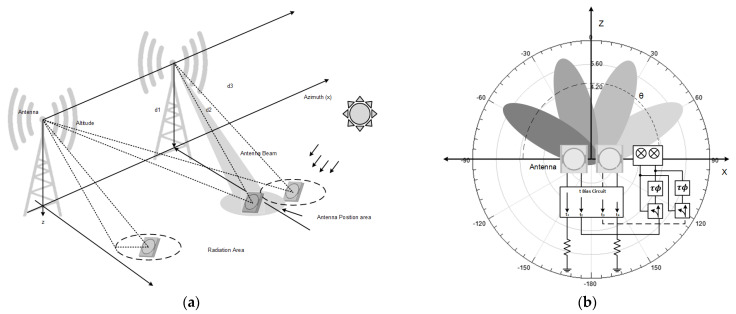
(**a**). Schematic analysis of antenna configuration on the wireless sensor (**b**). CP antenna adaptive analysis of the radiation pattern.

Based on the configuration in the wireless sensor communication system, it can be analyzed as an adaptive antenna system which has been confirmed to provide significant advantages on single-input single-output [38,39] or multiple-input multiple-output [40,41]. This analysis has additionally proven powerful for applications such as location-based services, positioning for tracking, and wireless sensor networks. The adaptive antenna system can accommodate multiple beams according to the antenna’s radiation polarization specifications. In Figure 18b, it is shown that the maximum main beam can be directed towards the targeted object while spatially reducing interference. It reduces power to unwanted areas using the Direction-of-arrival estimation algorithm analysis with the schematic analysis of the antenna configuration in Figure 18a [42,43]. Assume that the antenna analysis is positioned in planar conditions with specifications according to performance tests, namely sensitive to circular propagation wave polarization and directional radiation polarization.

The analysis of the Direction-of-arrival algorithm in the configuration of Figure 5 describes each antenna element and they receive a signal. Then perform a spatial calculation of the correlation matrix. A comprehensive analysis described the ability of the spatial calculations process in the sensor antenna configuration system to be empirically implemented into an adaptive circuit system [44,45]. The investigation is carried out by sending 1250 propagation signals at the azimuth of the transmission position. The first step is to determine the transmitted signal x(τ) with different beams N times. This method can provide a spatial variation of the object because the observations on the M signal are empirically represented as a steering vector α(*ϕ*). With this method, the received signal vector N × 1 one y(τ) can be defined as Formula (7).

Y(τ) = α(*Φ*) × (τ) + y(τ) (7)

With the symbol index accepted as τ, then at K × 1, where the element has a variance equal to (*ϕ*) is the steering vector K × 1 as an additive to the white Gaussian noise vector expressed by x(τ). Therefore, in the steering vector, every element of the nth antenna can be defined by Equation (8).

(8) $$\alpha_{n}\left(\phi \right)=\sqrt{P_{Gain}^{H}}\overset{M}{\underset{m=1}{\sum }}I_{m}e^{\left[j\left(m-1\right)x_{0}d\left(sin\left(\phi \right)-sin\left(\theta_{m}\right)\right)\right]}$$

The antenna coordinates at the transmission azimuth angle defined as a perpendicular direction to the multi-patch antenna as a planar angle representation, with the reference point distance on the multi-antenna in the form of *d*. The amplitude variance is the algorithm on the wireless sensor device at a 2.4 GHz RF antenna signal with *V*.

$$\left[\begin{array}{c} \begin{array}{c} Z_{1}\\ Z_{2} \end{array}\\ \begin{array}{c} Z_{3}\\ Z_{4} \end{array} \end{array}\right]=\left[\begin{array}{cc} \alpha \left(\phi_{1}\right) & \alpha \left(\theta \phi_{2}\right) \end{array}\begin{array}{c} \alpha \left(\phi_{3}\right)\alpha \left(\phi_{4}\right) \end{array}\right]\left[\begin{array}{c} \begin{array}{c} V_{1}e^{j\omega t}\\ V_{2}e^{j\omega t} \end{array}\\ \begin{array}{c} V_{3}e^{j\omega t}\\ V_{4}e^{j\omega t} \end{array} \end{array}\right]$$

A random amplitude is added to the *Z* matrix at the signal transmission source, denoted by *ψ*, where the value of *ψ* from the propagation source is expressed in the matrix *ψ*. Then, on the noise subspace analysis. Estimating the received signal’s covariance matrix is necessary to find the noise subspace steering matrix Â with the steering matrix of α(*ϕ*). Then, the data generated from the conjugate complex = Â × *ψ* are obtained in the form of ϰ* from the ensemble average $\bar{\varkappa_{1}\varkappa_{1}^{*}},\;\bar{\varkappa_{2}\varkappa_{2}^{*}},\;\bar{\varkappa_{3}\varkappa_{3}^{*}},\;\bar{\varkappa_{4}\varkappa_{4}^{*}}$. The other cross-correlation values in the signal assume the signal plane, from the input signal itself to be autocorrelated, on the covariance matrix of the input signal *ϰ*. Where ***Rxx*** is the estimated covariance matrix which can be expressed as ${Ř}_{xx}=\frac{1}{M_{s}}\sum_{\tau =1}^{M_{s}}y\left(\tau \right)\;y^{H}\left(\tau \right)$, where the number of samples is defined by *M_s_*. Then, the decomposition of the eigenvalues on the covariance matrix can be described using Equation (9).

(9) $$\bar{\varkappa \varkappa^{*}}=\left[\begin{array}{cccc} \bar{\varkappa_{1}\varkappa_{1}^{*}} & \bar{\varkappa_{1}\varkappa_{2}^{*}} & \bar{\varkappa_{1}\varkappa_{3}^{*}} & \bar{\varkappa_{1}\varkappa_{4}^{*}}\\ \bar{\varkappa_{2}\varkappa_{1}^{*}} & \bar{\varkappa_{2}\varkappa_{2}^{*}} & \bar{\varkappa_{2}\varkappa_{3}^{*}} & \bar{\varkappa_{2}\varkappa_{4}^{*}}\\ \bar{\varkappa_{3}\varkappa_{1}^{*}} & \bar{\varkappa_{3}\varkappa_{2}^{*}} & \bar{\varkappa_{3}\varkappa_{3}^{*}} & \bar{\varkappa_{3}\varkappa_{4}^{*}}\\ \bar{\varkappa_{4}\varkappa_{1}^{*}} & \bar{\varkappa_{4}\varkappa_{2}^{*}} & \bar{\varkappa_{4}\varkappa_{3}^{*}} & \bar{\varkappa_{4}\varkappa_{4}^{*}} \end{array}\right]=Â\;{Ř}_{xx}\;{Â}^{H}+{Â}_{\mathrm{Noise}}$$

With values Ř*_xx_* = Ê*_sg_* Â*_sg_* Ê*_sg_*^H^ + Ê*_ns_* Â*_ns_* Ê*_ns_*^H^, and with values Ê*_sg_* = [*ê*_1_, *ê*_1_, *…*, *ê_n_*] including the eigenvector estimates for the signal subspace; Â*_sg_* = diag[*â*_1_, *â*_2_, …, *â_n_*] is diagonal matrix of the largest estimated eigenvalues. This is with the following subspace eigenmatrix values:

$${Â}_{sg}=\left[\begin{array}{ccccc} 0.6457 & 0 & 0 & 0 & 0\\ 0 & 0.7292 & 0 & 0 & 0\\ 0 & 0 & 0.8580 & 0 & 0\\ 0 & 0 & 0 & \ddots & 0\\ 0 & 0 & 0 & \ldots & 34,442.4915 \end{array}\right]\approx \left[\begin{array}{ccccc} 1 & 0 & 0 & 0 & 0\\ 0 & 1 & 0 & 0 & 0\\ 0 & 1 & 1 & 0 & 0\\ 0 & 0 & 0 & \ddots & 0\\ 0 & 0 & 0 & \ldots & 1 \end{array}\right]$$

After estimating the noise subspace for each eigenvector, the classification multi-signal spectrum can finally be generated as Equation (10).

(10) $$P_{sg}\left(\varphi \right)=\frac{\alpha^{*}\left(\varphi \right)\alpha \left(\varphi \right)}{\alpha^{*}\left(\varphi \right){Ê}_{ns}{Ê}_{ns}^{*}\alpha \left(\varphi \right)}$$

**Figure 19:**
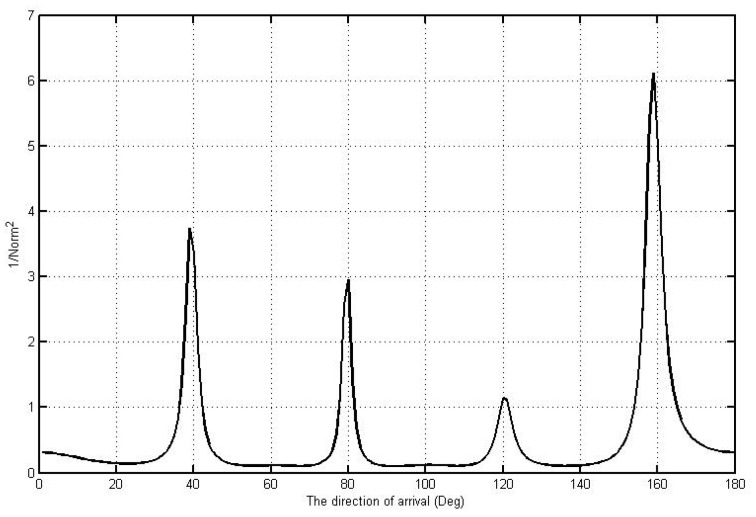
The spectrum of each signal arrival angle.

From Equation (10), distortion can occur. Sometimes a compromise is unavoidable in completing the direction of arrival with signal classification methods with ideal patterns when the actual signal pattern cannot be accessed. In this case, the decrease in error variance in the multi-signal classification is slightly different from the perfect condition, considering a distorted pattern which is involved in estimating the covariance matrix. Therefore, the signal and noise subspaces which can be seen in Figure 19 are the spectrum of each signal arrival angle.

The spectrum result can be partially analyzed considering the angle of origin of the signal as a whole in the signal sample, which is a transmission with the condition of random signal amplitude. The information signal spectrum is expressed in a graph with a normalized power level of 1/Norm^2^ for each signal spectrum. From each spectrum level, the degree of signal arrival can be represented at each spectrum peak with angle values of 40°, 80°, 120°, and 160° according to the angle in the sample signal transmission process with high accuracy under ideal conditions. Therefore, the algorithm can effectively solve the communication process on wireless sensors supported by wireless energy harvesting with electromagnetic harvesting and solar energy.

## 4. Conclusions

This study presents a new circuit configuration consisting of dual-feed antennas connected to a 2 × 4 asymmetric hybrid coupler and a multi-stage voltage doubler rectifier circuit. It has circular polarization sensitivity and is integrated with thin-film solar cells to accommodate energy harvesting and wireless sensors on communication networks simultaneously and independently without interference. This configuration has the specifications of two Microstrip antenna ports with an operating frequency of 2.4 GHz, Circular Polarization, high gain values, and directional radiation polarization. It has been integrated with thin-film solar cells and is connected to a solar electromagnetic energy harvesting network and wireless sensors on the network communication. This configuration has a good isolation value between the two feed ports (S11 or S21 < −15 dB) on the 2 × 4 asymmetric hybrid coupler to produce four outputs with a wave phase difference of 90 degrees. It is confirmed that arbitrary polarization of the input voltage multiplier waveform overcomes the uncertainty in the empirical communication environment. In testing the energy harvesting circuit, this system configuration produces a voltage power level for wireless sensors and low-power energy harvesting, with a maximum radiation value of 30 dBm with a parallel model integrated and a thin-film solar cell from the transmitter with a voltage of 1.3297 V with a compact composition. It is necessary to analyze circuit communication networks to adopt a wireless sensor strategy with multi-polarization sensitivity and integrated RF solar energy harvesting rather than a single-source method. It provides practical solutions for wireless sensor applications and independent energy harvesting.

## Figures and Tables

**Table 1:** Comparison of the CP Antenna with the 2 × 4 Hybrid Couple with Energy Harvesting and wireless sensing with previous research.

Ref	Freq (GHz)	S11	S21	Polarization	Eff %	Working Mode	Structure	Communication Analysis
[36]	1.8 and 2.45 GHz	−28dB	-	LP	43	Only energy harvesting	Multiple layers in the encapsulation	-
[37]	2.4 GHz	<−15 dB	<−15 dB	Dual CP	-	Communication and energy harvesting simultaneously	Two-layer Semiconductor	Multiple signal classification
[33]	1.7–2.6 GHz	<−10 dB	-	LP	-	Only energy harvesting	Single-layer Semiconductor	-
[34]	5.8 GHz	<−12.65 dB	<−12.04 dB	LP	51.73	Communication and energy harvesting separatedly	Single-layer Semiconductor	-
[35]	1.7–2.6 GHz	<−10 dB	-	LP	-	Only energy harvesting	Single-layer Semiconductor	-
This work	2.4 GHz	<−15 dB	<−15 dB	Dual CP	53	Communication and energy harvesting simultaneously or in independent operation	Two-layer Semiconductor	Multiple signal classification

## Data Availability

Not applicable.
